# 

**Published:** 1972-10

**Authors:** 


Bristol Medico-Chirurgical Journal Vol. 87 (iv) No.324

### Contents

Investigating Accidents in the Home ... ... 37
C. P. de Fonseka and J. L. Roberts

The Development and Usage of Clomiphene ... 53

Ingrid M. Bowdler

News and Notes ... ... ... ... 57

The Medical Library ... ... ... ... 58

Bristol Medico-Chirurgical Society ... ... 59

South-West Orthopaedic Club ... ... ... 59

Printed by F. Bailey & Son Ltd., Dursley, Glos.

4072

